# Evaluating and forecasting the associated main flavor components in Baijiu (Chinese distilled spirits) with alcohol metabolism and hangover symptoms through mice acute withdrawal model

**DOI:** 10.1002/fsn3.3064

**Published:** 2022-09-19

**Authors:** Jie Huangfu, Jun Lu, Changwen Li, Deliang Wang, Chunguang Luan, Xin Jiang, Tao Song, Wei Jiang, Xinlin Han, Jing Feng, Yanli Liu, Mengchao He

**Affiliations:** ^1^ China National Research Institute of Food & Fermentation Industries International Joint Research Center of Quality and Safety of Alcoholic Beverages Beijing China; ^2^ Guizhou Guotai Liquor Co., Ltd. Zunyi China; ^3^ Department of Biomedicine Beijing City University Beijing China

**Keywords:** alcohol metabolism and hangover symptoms, Baijiu (Chinese distilled spirits), main flavor compounds, mice acute withdrawal model

## Abstract

Over the past few decades, more alcohol‐problem concerns focused on reducing the risk of hangover caused by the alcoholic beverages over‐consumption. Chinese distilled spirits (Baijiu) is one of the most favorite alcoholic beverages. The intention of this study is to explore the associations of main flavor components in Baijiu and hangover symptoms using mice acute alcohol withdrawal model. The behaviors of each mouse were assessed by open‐field tests using separate groups of mice with the treatment of sauce‐aroma Baijiu, light‐aroma Baijiu, strong‐aroma Baijiu, pure alcohol, and distilled water, respectively. The behavioral data including total move distance and immobile time were used as indicators for the evaluation of the liquor intoxicating effects. Alcohol and acetaldehyde concentrations in mice plasma and the neurotransmitter contents of GABA and Glu in mice cerebellum were detected afterward. The results showed that the mice with the treatment of Baijiu samples displayed unusual exciting behaviors including increased alcohol metabolization with alleviating drunken and hangover symptoms, compared with that of pure alcohol control groups after 2–4 h. Moreover, the sauce‐aroma Baijiu treatment group showed lessening intoxicated symptoms than those of light‐aroma Baijiu and strong‐aroma Baijiu. In addition, there were significant differences between Baijiu and pure alcohol treatment groups at the inhibitory neurotransmitter GABAergic levels and its receptor GABA‐AR1 activating levels in the mice neuron cells. Furthermore, the Principal Component Analysis (PCA) analysis inferred that the flavor compounds acetic acid, ethyl acetate, ethyl lactate, and 1‐propanol in the sauce‐aroma Baijiu were played the major roles in the drunk behaviors that caused by the hangover. While, the acetic acid in the sauce‐aroma Baijiu was speculated as a major flavor component to accelerate the alcohol metabolism and retard hangover symptoms.

## Graphical abstract

Chinese distilled spirits (Baijiu) is one of the most favourite alcohol beverages. The flavor quality of Baijiu is supposed to be one of the key factors which caused the hangover. However, little systematic study has been done to estimate the flavors effects on the hangover in the different aroma‐type of Baijiu. To resolve this problem, we established the mice acute withdrawal model to investigate the associations with main flavor compounds in strong aroma, light aroma, sauce aroma Baijiu on alcohol metabolism and hangover signs, respectively. The notably alleviated hangover symptoms with the increase in the locomotion activity, the decrease in the accumulation of blood alcohol and acetaldehyde of mice, and the alteration of neurotransmitter changes were apparently found in the Baijiu treated group, in particular sauce aroma Baijiu, than that in the absolute alcohol treatment group. the Principal Component Analysis (PCA) analysis inferred that the flavor compounds ethyl acetate, ethyl lactate, acetic acid, and 1‐propanol in the sauce‐aroma Baijiu were played the major roles in the drunk behaviors that caused by the hangover. While, the acetic acid in the sauce‐aroma Baijiu was speculated as a major flavor component to accelerate the alcohol metabolism and retard hangover symptoms.

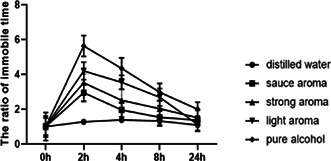

## 1 INTRODUCTION

Baijiu (Chinese distilled spirits or Chinese liquor) is normally fermented and distilled from sorghum alone or a mixture of corn, rice, wheat, peas, millet, and sorghum, which is famous for its brewing history (Liu & Sun., 2018). The three important typical flavor Baijiu types are sauce‐aroma (Jiangxiangxing), strong‐aroma (Nongxiangxing), and light‐aroma (Qingxiangxing), which are more widely distributed in Guizhou province, Sichuan, Jiangsu and Anhui province, Shanxi and Hebei province, respectively (Wei et al., 2020). Baijiu is reported to contain more than 2047 kinds of flavor compounds, which are produced from the mixed fermentation and distillation of microbial starters “jiuqu” and grains (Sun et al., 2021). Alcohols, esters, aldehydes, and acids are the four abundant kinds of flavors in Baijiu. The category and content of these substances vary greatly in different Baijiu aroma types (Cai et al., 2019). Primarily, in strong‐aroma Baijiu, the concentration of ethyl hexanoate and hexanoic acid is higher than that of other aroma compounds (Fan & Qian, 2006). In light‐aroma Baijiu, ethyl acetate is the main flavor substance followed by ethyl lactate (Gao et al., 2014; Niu et al., 2017). The flavor compounds are more diverse and complicated in sauce‐aroma Baijiu. The organic acids content, such as the acetic and lactic acid, display the highest levels in the than other Baijiu aroma types (Fan et al., 2011).

The alcohol hangover has substantial economic and health consequences. The obvious symptoms are dullness, weakness, incoordination, fatigue, sleepiness, thirst, headache, and mood change (Penning et al., 2010; 2013). Asian may experience more severe hangover symptoms, correlating to some extent with the presence of the aldehyde dehydrogenase (ALDH2) gene genetic variants (Wall et al., 2000). The alcohol and its metabolites acetaldehyde, immune, hormonal, and dehydration‐related factors of physiological changes and cognitive functions are the determinants of hangover severity (Mackus et al., 2020). The congeners such as methanol, l‐propanol, 1‐butanol, 2‐butanol, iso‐butanol, 2‐methyl‐butanol, 3‐methyl‐butanol in alcoholic beverages were suggested to affect hangover severity (Rohsenow & Howland., 2010). However, alcohols without congeners were also found to produce hangovers (Rohsenow et al., 2010).

In China, nearly 20% of drinkers are described to not experience hangover symptoms despite heavy drinking of Baijiu. They attributed the reason for the relating to the different flavor compounds changes in production processing of Chinese and non‐Chinese liquors, such as fermentation, distillation, aging, blending processes, raw materials, environmental conditions, brewing microorganisms, and fermentation starters. Furthermore, the balance and coordination of these volatile compounds not only affect the sensory quality but also affect the symptomatic relief of hangover. Nevertheless, current data comparing alcoholic beverages are limited, and little evidence indicates any effective components to prevent the hangover symptoms in Baijiu.

The similarity in biology and symptoms between the animal model and the human hangovers is generally acknowledged as previous studies described (Lucas et al., 2018). In this work, the mice acute withdrawal model was established to investigate the associations with main flavor compounds in strong‐aroma Baijiu, light‐aroma Baijiu, sauce‐aroma Baijiu, and pure alcohol on alcohol metabolism and hangover symptoms.

## 2 MATERIALS AND METHODS

### 2.1 Baijiu samples

All the Baijiu samples were commercial liquor products that were purchased from the local markets (Beijing, China). The information for the Baijiu samples used in this study was listed in Table 1. The pure alcohol sample for the same ethanol content was prepared by the solution of commercial vodka (Spirytus rektyfikowany, Poland, 96%, ABV) with distilled water.

**TABLE 1:** Information for the Baijiu samples

Baijiu aroma type	Origin	Ethanol content
sauce‐aroma	produced by Guotai Co. Ltd., Guizhou, China	53%, ABV
light‐aroma	produced by Fenjiu Co. Ltd., Shanxi, China	53%, ABV
strong‐aroma	produced by Luzhoulaojiao Co. Ltd., Sichuan, China	53%, ABV

### 2.2 Animals

The animals used in this study were C57BL/6J wide‐type (WT) male mice with 65 days of age and an average body weight of 20 g, which were housed under standardized condition. The mice were randomly divided into five groups: Baijiu treatment, purified alcohol treatment, and distilled water treatment (*n* = 6, for each group). Each animal was treated with Baijiu and pure alcohol samples at the same dosage (0.6 g of ethanol per 100 g body weight) or a matched volume of distilled water through an infusion method using 1‐ml intragastric syringes. All in vivo testing was performed in accordance with the China Laboratory Animal Guideline for Ethical Review of Animal Welfare (GB/T 35892‐2018) and approved by China National Research Institute of Food & Fermentation Industries’ Animal Welfare and Ethical Review Body (CNRIFFI 2020‐w‐005).

### 2.3 Open‐field behavioral assessment

In this study, mice after‐drinking behaviors assessment was utilized by Open‐field. Mice were individually placed in a 30 cm × 70 cm × 47 cm arena with opaque walls. Sessions were video recorded and analyzed using ANY‐maze video tracking software (Stoelting, Wood Dale, IL).

### 2.4 Blood alcohol concentration determination

After the locomotor activity experiment, the animals were sacrificed directly. The blood (~0.5 ml) was then extracted via cardiac puncture. The blood was collected in sodium fluoride containing tubes to stop further alcohol metabolism. Then, these blood samples were centrifuged at 840 × g/rcf for 10 min, the serum was collected and aliquoted. The alcohol and its metabolites acetaldehyde concentration in the plasma were determined using headspace gas chromatography methods previously reported (Heit et al., 2016).

### 2.5 Neurotransmitter contents and immunofluorescence analysis

The Baijiu, distilled water, and pure alcohol were given to the mice, respectively. Two hours later, the cerebellums in the mice brain were excised. Homogenates were generated using homogenizer and RIPA buffer. The neurotransmitter contents of GABA and Glu were measured using commercial ELISA kits (Shanghai enzyme‐linked immunosorbent biotechnology co., Ltd, China). To determine the GABA extents in the neuron cells activation level, immunofluorescence analysis was conducted using surface GABABR1 staining (1/200 dilution; Abcam, ab55051). Image analysis platform was used to analyze the features.

### 2.6 Baijiu flavor analysis

Volatile flavors in Baijiu including fusel alcohols, esters, and aldehydes were analyzed by the HS‐SPME GC–MS methods according Chinese standard (GB/T10345). The gas chromatograph (Clarus 600, PerkinElemer, MA, USA) was equipped with a flame ionization detector (GC‐FID). The CP‐Wax 57CB column (50 m × 0.25 mm I. D. × 0.20 μm df) was purchased from Agilent Technologies (Wilmington, DE, USA) using nitrogen gas (>99.999%) at a constant flow rate of 1.0 ml/min. The temperature of the column was maintained at 40°C for 3 min, then was increased to 80°C at 4°C/min, and held at 130°C for 9 min followed by an increase to 230°C at 15°C/min for 15 min. The internal standard was a mixture solution of analytical n‐butyl acetate, 2‐ethyl butyric acid, and methyl‐2‐butanol. The Baijiu samples were injected with a volume of 2% internal standard solution. Each expected analytical aroma compound of the standard gradient concentrations was prepared. For HS‐SPME sampling, an MPS‐2 autosampler (Gerstel, Mülheim, Germany) was used to extract volatile aromatic compounds, with a sample volume of 1 μl. The identification and analysis of organic acids were performed on a ICS‐3000 ion‐chromatography system (Dionex Corporation, American). The Ionpac AS11‐HC type separation column (250 mm × 4 mm) and Ionpac AG11‐HC type guard column (50 mm × 4 mm) were used, with a sample volume of 25 μl. Compounds were identified by comparing the standard curve with those of pure standards under the same conditions. The identification and quantification were carried out by comparing the retention times and standard curves (*R* ^2^ > 0.99) with those of known pure compounds.

### 2.7 Statistical analysis and multiple linear regression analysis

Data were analyzed and graphed using Prism version 8.3.1 (GraphPad Software, Inc. La Jolla, CA). For all experiments, a two‐Way ANOVA with Dunnett's multiple comparisons test was used to determine differences by SPSS 22.0 software. Data were considered to be statistically significant at *p* < .05. Principal Component Analysis (PCA) was applied to the data set to visualize the association of the drunkenness degree behavioral indicators and the Baijiu flavors source levels using SPSS 22.0 software.

## 3 RESULTS AND DISCUSSION

### 3.1 Open‐field behavioral assessment in alcoholized mice

**FIGURE 1:**
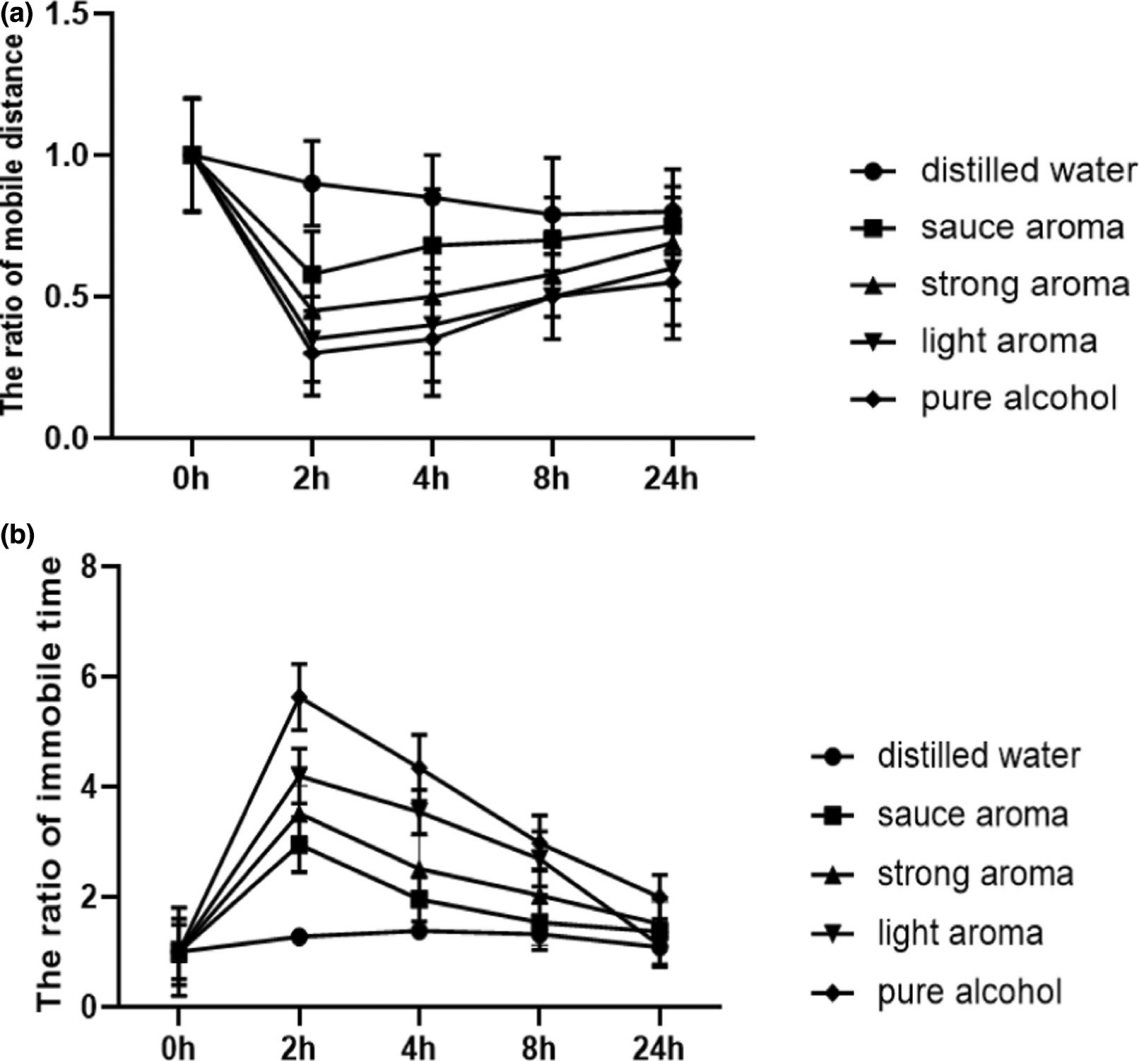
The ratios of the C57BL6 mice (*n* = 5) behavioral parameters at 2, 4, 8, and 24 h after intragastric administration to that of 0 h before intragastric administration with sauce‐aroma Baijiu type, strong‐aroma Baijiu type, light‐aroma Baijiu type, pure alcohol, and distilled water, respectively. (Figure 1a, mobile distance (m ± SEM), Figure 1b, immobile time (s ± SEM))

Behavioral changes are the key measurement criteria of evaluating different aspects of alcohol hangover in rodents (Karadayian & Cutrera, 2013; Lucas et al., 2018). The open‐field test has several merits in this regard as it monitors rodent behaviors in a novel environment with the ability to simultaneously measure exploration, anxiety‐like behaviors, and general locomotion. These time course models can be used to definitively correlate variables of interest to hangover severity over time (Je et al., 2021). This type of model could provide valuable insight into how changing biochemical markers may influence or contribute to alcohol hangover (Bustamante et al., 2012; Juarez et al., 2017). In this study, for the majority of behavioral measures considered, the covered locomotion distance and immobile time persisted for 24 h were the major measurement of after‐drinking behaviors in mice. The ratios of after‐drinking motor performance to that of before‐drinking were displayed in Figure 1. The Baijiu treatment group differed significantly from pure alcohol and distilled water at 2–8 h, in particular the sauce‐aroma Baijiu (*p <* .05). Compared with pure alcohol treatment group, all the Baijiu aroma types treatment groups of mice displayed obvious longer mobile distance and shorter moving time. The sauce‐aroma Baijiu treatment mice group showed lessening immobile time than those of light‐aroma Baijiu and strong‐aroma Baijiu. The results indicated that Baijiu, especially sauce‐aroma Baijiu, might alleviate alcohol hangover severity of behavioral influences than that of pure alcohol.

### 3.2 Blood alcohol and acetaldehyde concentrations assessment in alcoholized mice

**FIGURE 2:**
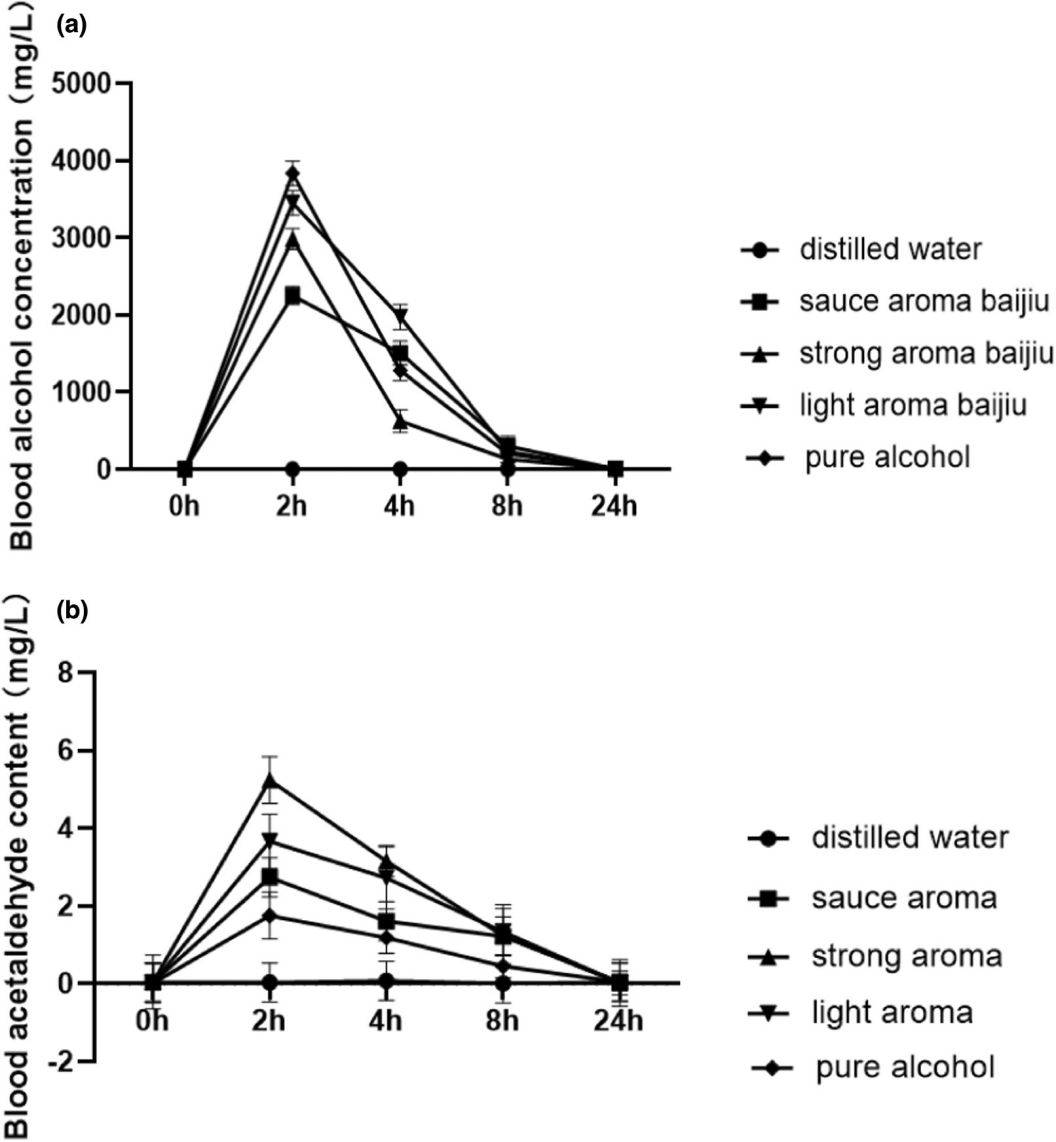
Measures of blood alcohol and acetaldehyde concentration analyzed 2, 4, 8, and 24 h after C57BL6 mice (*n* = 5) were dosed intragastrically with Baijiu, pure alcohol, and distilled water, respectively. (Figure 2a, blood alcohol concentration, Figure 2b, blood acetaldehyde concentration)

The alcohol hangover refers to the combination of negative mental and physical symptoms which can be experienced after a single episode of alcohol consumption, starting when blood alcohol concentration (BAC) approaches zero (Verster et al., 2020). The rate of alcohol metabolism proposed to be an important determinant of hangover severity (Mackus et al., 2020). Measuring alcohol and its metabolites concentration to indicate a hangover state was a common method used in the alcohol hangover model (Penning et al., 2010; Tipple et al., 2016; Palmer et al., 2019). In this study, the blood alcohol and acetaldehyde concentrations were determined 2, 4, 8, and 24 h after C57BL6 mice were dosed intragastrically with pure alcohol, distilled water, and different Baijiu aroma types (Figure 2). The results demonstrated that at 24‐h post‐administration with all the alcoholic beverages, the BAC was returning to zero. Notably, the mice behavioral data displayed that the pure alcohol and Baijiu treatment groups did not return to normal at this time point, and still experienced a movement retardation than the water control. After intragastric filling for 2–4 h, the BAC for sauce‐aroma Baijiu treatment group had statistically significant difference from that for other alcohol beverage treatment groups (*p <* .05). The treatment of the sauce‐aroma Baijiu group exhibited the lowest BAC peak; however, the pure alcohol treatment group presented the highest BAC peak. In this study, acetaldehyde was difficult to measure in the blood at 24‐h post‐alcohol administration. Meanwhile, there was a statistically significant difference of acetaldehyde content in mice blood of dosing intragastrically with different Baijiu types after 2–4 h (*p <* .05). Interestingly, the sauce‐aroma Baijiu treatment group with the rapid alcohol metabolism did not display the high acetaldehyde conversion rate. The flavor compounds in the sauce‐aroma Baijiu was speculated to reduce the negative effect on adverse symptoms experienced during hangover through the synergistic efficiency mechanism with pure alcohol.

### 3.3 Neurotransmitter expression analysis

**FIGURE 3:**
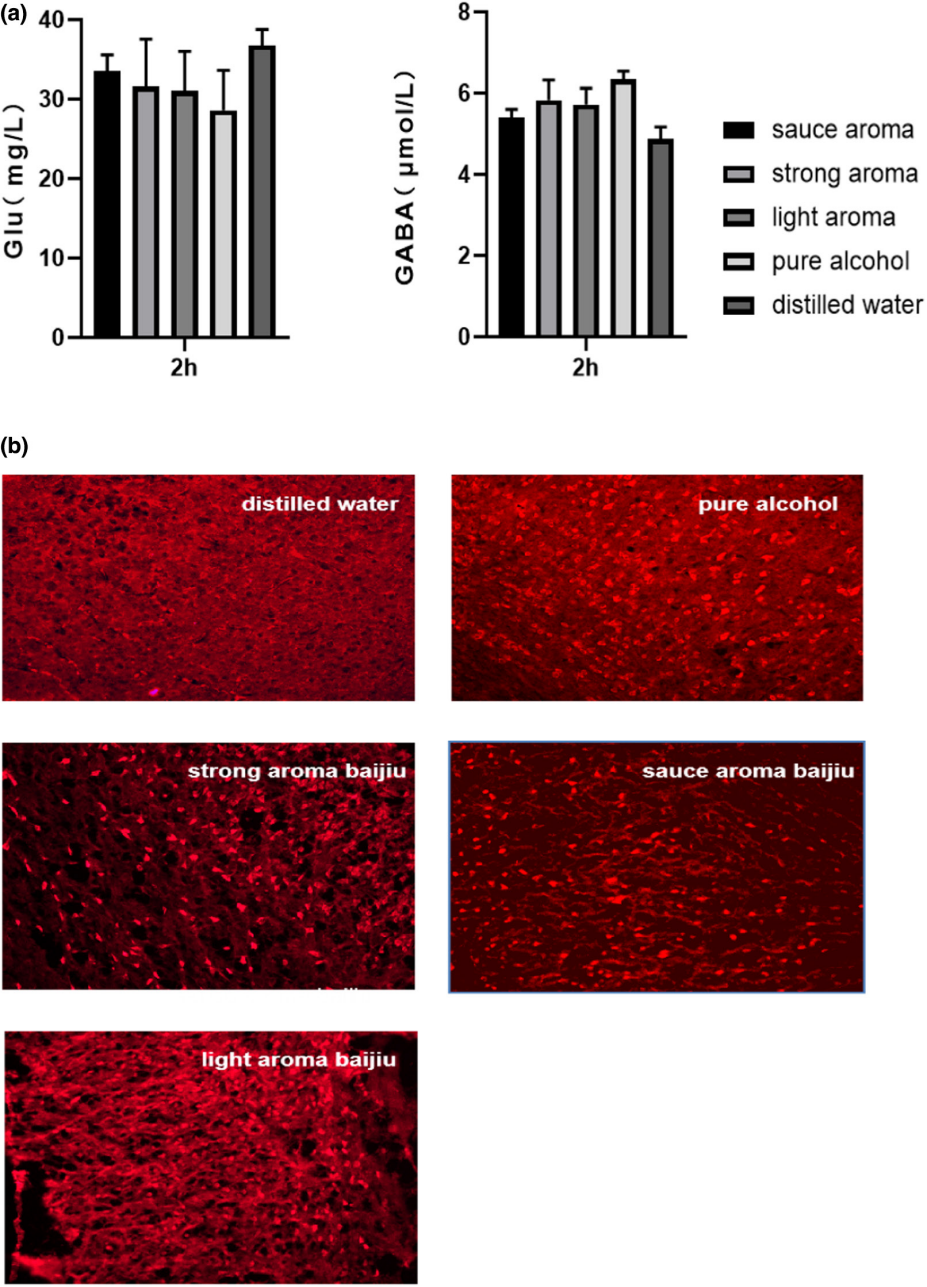
(a) Measures of neurotransmitter *Glu* and *GABA* levels in the cerebellum of mice brain analyzed 2 h after C57BL6 mice (*n* = 5) were dosed intragastrically with Baijiu, pure alcohol, and distilled water, respectively. (b) GABA‐AR expression levels in the neuron cells of cerebellum after C57BL6 mice were dosed intragastrically with Baijiu, pure alcohol, and distilled water after 2 h, respectively.

Alcohol hangover is influenced by multiple factors, including notably ethanol metabolites, neurotransmitter alterations, inflammatory markers, and mitochondrial dysfunction (Van‐de‐Loo et al., 2010; Palmer et al., 2019). The neurotransmitters such as gamma amino butyric acid (GABA), glutamate (Glu), dopamine (DA), serotonin (5‐HT), and dopamine (DA) were reported as the dominantly affected neurotransmitters by acute alcohol consumption (Chastain, 2006; Palmer et al., 2019). In this study, the levels of GABA and Glu in the cerebellum of mice brain following intragastric administration of pure alcohol, distilled water, and Baijiu at the time of 2 h were analyzed, respectively. The data were illustrated in Figure 3a. The results showed that in pure alcohol and Baijiu administration groups the inhibitory GABAergic levels were significantly reduced and the excitatory glutamatergic levels were significantly elevated than that of water control treatment group (*p <* .05). This phenomenon was in accord with the preliminary conclusions that alcohol exposure might alter the balance between GABA and Glu neurotransmission and cause the changes of behaviors, in particular increased distance and decreased mobile time (Williams et al., 2018). Moreover, at the GABA‐A receptor 1 (GABA‐AR1) activation levels in the neurons cells of mice cerebellum, there was significant difference between Baijiu administration groups and pure alcohol treatment groups (Figure 3b). ALDH2 in the brain cerebellum cells controls the production, cellular, and behavioral effects of alcohol metabolites in a brain‐region‐specific manner through mediating ethanol‐acetate induced elevation of GABA levels and enhancement of GABA‐A receptor function (Jin et al., 2021). These results demonstrated that Baijiu was notably differ from pure alcohol on the contribution of neuronal ALDH2 to ethanol‐induced effects. However, the difference between GABA receptor sensitivity in different Baijiu aroma type treatment groups was not obvious.

### 3.4 Main flavor compounds in Baijiu association analysis

**FIGURE 4:**
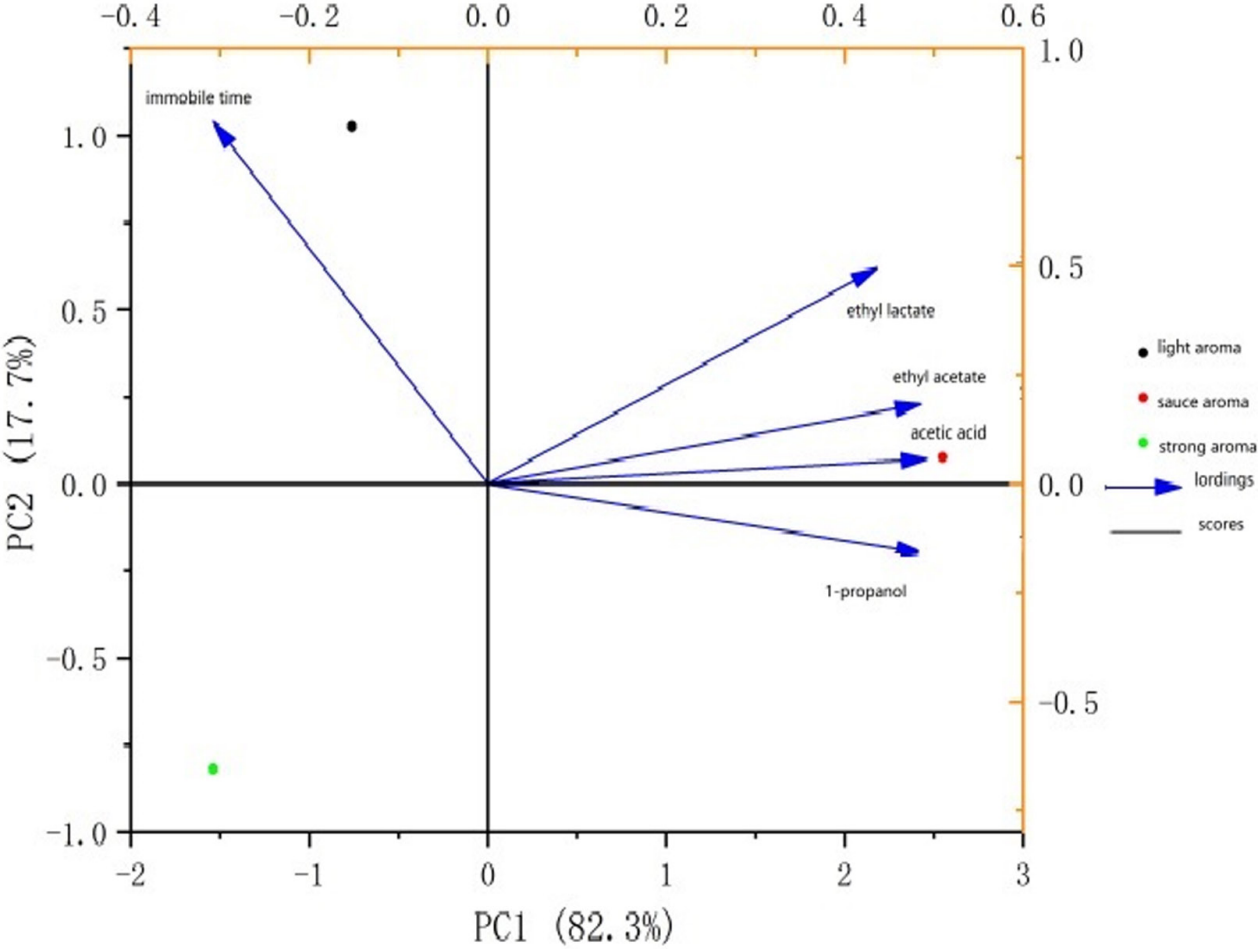
Principal component analysis for the association of main flavor Baijiu components and locomotion behavior immobile time at 2 h after C57BL6 mice (*n* = 5) were dosed intragastrically with Baijiu.

  Some compounds within alcoholic beverages are produced during distillation and fermentation, which may contribute to the severity of hangover induced by alcohol (Rohsenow et al., 2010). The determinations of main flavor compounds in sauce‐aroma Baijiu, light‐aroma Baijiu, and strong‐aroma Baijiu were demonstrated in Table 2. The results demonstrated that the contents of main flavor components, namely ethyl acetate, ethyl lactate, acetic acid, and 1‐propanol, up to more than 1000 mg/L in sauce‐aroma Baijiu, far more than light‐aroma Baijiu and strong‐aroma Baijiu. It was previously reported that fusel alcohols and aldehydes in alcoholic beverages, such as whisky, brandy, wine, beer, and Chinese Baijiu, had performance‐enhancing effects on the intoxicating degree (Hisako et al., 2003; Wu et al., 2016; Xiao et al., 2018). However, the functions of esters and organic acids in Baijiu on alcoholism have not been proved. To find the existing relationship between negative behaviors data in experimental drunken mice post‐alcohol administration at 2‐h time point and the contents of the mentioned three main flavor components, the Principal Component Analysis (PCA) was used in this study. These two methods are simple algorithms widely used to explore the correlation between independent variables and the dependent variable (Liu et al., 2020; Wu et al., 2016). The PCA analysis provided insight into the effects of main flavors ethyl acetate, ethyl lactate, acetic acid, and 1‐propanol in sauce‐aroma Baijiu on the immobile moving time of mice behavior after acute alcohol consumption of 2h (Figure 4a). The cumulative variance contribution rate of the first PCA and the second principal component was 100% (PC1 82.3%, PC2 17.7%). By either PC1 or PC2, the light aroma Baijiu was clustered in the second quadrant, the strong aroma Baijiu was clustered in the third quadrant, the sauce aroma Baijiu was clustered in the fourth quadrant, and the immobile moving time of drunken mice behavior was mainly related to the light‐aroma Baijiu. The acetic acid was mainly related to the sauce‐aroma Baijiu and had a further relationship from the immobile moving time, which was predicted to be positively affected on reducing hangover severity.

**TABLE 2:** Main flavor compounds in Baijiu

Flavor compounds in (mg/L)	Sauce‐aroma	Light‐aroma	Strong‐aroma
Fusel alcohols			
Methanol	112.5 ± 4.3	114.1 ± 3.5	65.6 ± 1.4
2‐butanol	52.3 ± 4.5	1.9 ± 1.2	12.4 ± 1.1
1‐propanol	1194.6 ± 6.9	140.3 ± 1.6	87.4 ± 1.3
2‐methylpropanol	162.5 ± 5.2	106.8 ± 2.6	24.1 ± 1.5
2‐pentanol	4.08 ± 1.2	N/D	3.3 ± 0.9
1‐butanol	126.8 ± 2.5	14.4 ± 2.5	90.4 ± 1.2
(2S)‐2‐methyl‐1‐butanol	80.2 ± 8.3	56.3 ± 1.3	14.7 ± 0.6
3‐methyl‐1‐butanol	264.2 ± 2.5	260.8 ± 4.2	65.0 ± 1.3
1‐pentanol	8.7 ± 1.4	N/D	4.5 ± 0.2
1‐hexanol	17.8 ± 1.5	3.1 ± 0.3	29.4 ± 1.1
2,3‐butanediol (left spin)	53.9 ± 2.5	18.1 ± 0.5	10.7 ± 1.4
2,3‐butanediol (inner spin)	35.8 ± 4.3	6.0 ± 0.3	5.2 ± 1.2
1,2‐propanediol	144.1 ± 2.5	N/D	8.1 ± 0.2
2‐phenylethanol	15.9 ± 1.2	4.8 ± 0.4	2.2 ± 0.1
Esters			
Ethyl formate	53.5 ± 3.4	4.1 ± 0.5	19.3 ± 0.2
Ethyl acetate	2699.3 ± 5.2	1345.3 ± 4.2	949.1 ± 4.2
Ethyl butanoate	103.1 ± 4.7	N/D	207.3 ± 1.3
Ethyl 3‐methylbutanoate	8.1 ± 1.5	N/D	2.5 ± 0.4
3‐methylbutyl acetate	4.7 ± 1.4	N/D	N/D
Ethyl pentanoate	18.7 ± 1.3	4.6 ± 1.4	29.2 ± 1.3
Ethyl hexanoate	62.9 ± 1.2	N/D	1527.3 ± 2.5
Ethyl heptanoate	2.1 ± 2.3	N/D	12.6 ± 1.3
Ethyl lactate	1090.9 ± 15.3	1003.9 ± 4.2	500.3 ± 1.5
Ethyl octanoate	8.1 ± 0.3	1.7 ± 0.2	11.1 ± 1.4
3‐methylbutyl hexanoate	N/D	1.5 ± 0.4	N/D
Diethyl butanedioate	3.8 ± 0.3	9.9 ± 1.3	N/D
Ethyl dodecanoate	3.1 ± 0.4	41.7 ± 5.2	N/D
Ethyl tetradecanoate	1.3 ± 0.5	N/D	N/D
Ethyl hexadecanoate	30.6 ± 1.3	N/D	N/D
Ethyl (Z)‐octadec‐9‐enoate	12.7 ± 2.5	N/D	N/D
Ethyl (9Z,12Z)‐octadeca‐9,12‐dienoate	18.0 ± 1.6	N/D	N/D
Aldehydes			
Acetaldehyde	479.9 ± 4.2	179.8 ± 3.2	197.8 ± 2.4
Propanal	6.2 ± 0.4	N/D	N/D
2‐methylpropanal	17.7 ± 1.2	N/D	3.06 ± 0.3
1,1‐diethoxyethane	332.0 ± 5.2	118.6 ± 1.3	112.8 ± 1.5
3‐methylbutanal	78.2 ± 3.7	13.7 ± 0.6	13.1 ± 0.4
Furan‐2‐carbaldehyde	326.5 ± 3.2	1.7 ± 0.2	12.5 ± 0.2
Benzaldehyde	3.0 ± 0.2	N/D	N/D
Organic acids			
Lactic acid	691.3 ± 2.3	433.9 ± 3.2	221.2 ± 1.3
Acetic acid	2043.1 ± 11.2	1370.1 ± 5.4	1085.8 ± 3.6
Propanoic acid	60.5 ± 2.3	N/D	6.7 ± 4.2
Formic acid	34.75 ± 2.8	N/D	16.8 ± 1.5
Isobutyric acid	4.9 ± 0.4	N/D	5.6 ± 1.3
Butanoic acid	42.3 ± 2.6	N/D	175.1 ± 1.2
Hexanoic acid	20.1 ± 1.5	N/D	2083.5 ± 4.3
Sulfate radical	64.0 ± 2.4	N/D	N/D

*Note*: Data are expressed as mean ± standard deviation.

Abbreviation: N/D, Not Detected.

Sauce‐aroma Baijiu is becoming increasingly accepted in Chinese for the moment. Many drinkers generally reflected that there were little hangover symptoms after heavy drinking of sauce‐aroma Baijiu. Several previous studies speculated positive correlation between the active no‐alcoholic components such as pyrazine and their pharmacological effects in Sauce‐aroma Baijiu (Zhou et al., 2019). However, these active components were low contained extremely in Baijiu (Zhou et al., 2020). The literature has not yet substantiated their positive impacts by experiments in the whole alcohol beverages intake currently. Baijiu is a distilled liquor, but its production combines the two distinctive processes of fermentation and distillation. These non‐volatile acids in Baijiu were from the fermented grains by acidogenic bacteria (Liu & Sun, 2018). Most studies illustrated that the lower content of acids in the no‐alcoholic beverages and fermented alcoholic beverages such as wine or beer have positive impacts on health (Valentine et al., 2021; Rhee et al., 2011). This study first demonstrated that the acids in sauce‐aroma Baijiu were much more than both light‐aroma and strong‐aroma Baijiu, presuming it probably had positive effect on reducing hangover severity. The result might be inferred to instructive significance for the improvement of hangover severity from the brewing and blending process in Baijiu.

## 4 CONCLUSIONS

Alcohol hangover is commonly considered as a public health concern in the world. General Chinese population may experience more severe symptoms compared to European and African ancestries, due to the marked lower clearance rate of alcohol. The two genetic variants in the Chinese alcohol metabolizing‐related genes ALDH2 (rs671) and ADH1B (rs1229984 and rs2066702) that greatly alter alcohol metabolism and had a higher risk of acetaldehyde accumulation and esophageal cancer (Iona et al., 2019). In the last 20 years, the alcohol consumption continued to rise in China with the weak awareness of excessive drinking. Three dominant aroma types of Chinese Baijiu, namely, strong‐aroma, light‐aroma, sauce‐aroma, which comprised more than 80% of total Chinese Baijiu output. So far, however, there was little discussion about the differences in hangover effects caused by the three dominant Baijiu. The main aim of this investigation was to assess the impacts on the susceptibility of alcohol hangover by strong‐aroma, light‐aroma, sauce‐aroma Baijiu, and pure alcohol, to determine the associated flavor components that might affect synergically on alcohol metabolism through mice acute alcohol withdrawal model. The notably alleviated hangover symptoms with the increase in the locomotion activity, the decrease in the accumulation of blood alcohol and acetaldehyde of mice, and the alteration of neurotransmitter changes were apparently found in the Baijiu treatment group, than that in the absolute alcohol treatment group. The neurotransmitter alterations of GABA and Glu were displayed to correlate with intensity of behavior symptoms in the pure alcohol treatment group. The PCA analysis suggested that the main flavor compounds acetic acid in the sauce‐aroma Baijiu had positive effects on preventing alcohol hangover for motor performance. Furthermore, the mice that were given the sauce‐aroma Baijiu exhibited lessening intoxicated symptoms than those of light‐aroma and strong‐aroma Baijiu. The results suggested that the organic acids in the sauce‐aroma Baijiu was of great importance to accelerate the alcohol metabolism. Previous study had revealed that the organic acid fraction in Japanese Sake, particularly L‐lactic acid, gluconic acid, and pyruvic acid, played important roles in potentiating the GABAA receptor‐mediated response which led to significant anxiolytic effects (Hanae et al., 2017). Notably, the role of GABA in alcohol responsiveness has been proved in animal models. However, it is not obvious whether the organic acid fraction in the Baijiu directly mediates the response. To elucidate it, further analysis is required.

## AUTHORS’ CONTRIBUTIONS

The manuscript was written through contributions of all authors. All authors have given approval to the final version of the manuscript.

## FUNDING INFORMATION

This study was supported by China alcoholic drinks association Baijiu foundation projects (20210408); Guizhou and Zunyi key research projects (GY2020‐40; 2020‐2Y045); Intergovernmental international key research cooperation projects (2021YFE0192000).

## CONFLICT OF INTEREST

The authors declare no conflict of interest.

## Data Availability

The data that support the findings of this study are available on request from the corresponding author upon reasonable request.
